# Data on the purification and crystallization of the loss-of-function von Willebrand disease variant (p.Gly1324Ser) of the von Willebrand factor A1 domain

**DOI:** 10.1016/j.dib.2016.05.004

**Published:** 2016-05-10

**Authors:** James C. Campbell, Alexander Tischer, Venkata Machha, Laurie Moon-Tasson, Banumathi Sankaran, Choel Kim, Matthew Auton

**Affiliations:** a Structural and Computational Biology and Molecular Biophysics Program, Baylor College of Medicine, Houston, TX, USA; b Division of Hematology, Departments of Internal Medicine and Biochemistry and Molecular Biology, Mayo Clinic, Rochester, MN, USA; c Berkeley Center for Structural Biology, Lawrence Berkeley National Laboratory, 1 Cyclotron Road, BLDG 6R2100, Berkeley, CA, USA; d Department of Pharmacology, Baylor College of Medicine, Houston, TX, USA; e Verna and Marrs McLean Department of Biochemistry and Molecular Biology, Baylor College of Medicine, Houston, TX, USA

**Keywords:** von Willebrand factor, von Willebrand disease, Protein crystallization, Platelet adhesion

## Abstract

von Willebrand factor׳s (VWF) primary hemostatic responsibility is to deposit platelets at sites of vascular injury to prevent bleeding. This function is mediated by the interaction between the VWF A1 domain and the constitutively active platelet receptor, GPIbα. The crystal structure of the A1 domain harboring the von Willebrand disease (vWD) type 2M mutation p.Gly1324Ser has been recently published in the Journal of Biological Chemistry describing its effect on the function and structural stability of the A1 domain of VWF, “Mutational constraints on local unfolding inhibit the rheological adaptation of von Willebrand factor” [1]. The mutation introduces a side chain that thermodynamically stabilizes the domain by reducing the overall flexibility of the A1–GPIbα binding interface resulting in loss-of-function and bleeding due to the inability of A1 to adapt to a binding competent conformation under the rheological shear stress blood flow.

In this data article we describe the production, quality control and crystallization of the p.Gly1324Ser vWD variant of the A1 domain of VWF. p.Gly1324Ser A1 was expressed in *Escherichia coli* as insoluble inclusion bodies. After the preparation of the inclusion bodies, the protein was solubilized, refolded, purified by affinity chromatography and crystallized. The crystal structure of the p.Gly1324Ser mutant of the A1 domain is deposited at the Protein Data Bank PDB: 5BV8

**Specifications table**

**Table:** 

Subject area	Biology
More specific subject area	Structural biology
Type of data	Figures, tables, graphs
How data was acquired	Analytical gel filtration and Reverse phase HPLC (both performed on Beckman System Gold Analytical HPLC Systems), X-ray diffraction was performed at the Advanced Light Source beamline 5.0.1 using a ADSC Q315R detector. X-ray data was processed using iMOSFILM. The model was refined using the PHENIX software package and build using Coot.
Data format	Processed and analyzed
Experimental factors	VWF A1-p.Gly1324Ser was expressed in *E. coli* as inclusion bodies, processed, solubilized and refolded. The protein was purified to homogeneity through Ni-NTA and Heparin affinity chromatography. VWF A1-p.Gly1324Ser purity and absence of aggregates were confirmed by size exclusion and reverse phase chromatography.
Experimental features	The crystal structure of p.Gly1324Ser was determined via X-ray crystallography.
Data source location	Advanced Light Source, Berkley, California
Data accessibility	Crystallographic data within this article were deposited in the Protein Data Bank, PDB: 5BV8.

**Value of the data**

• This is the first crystal structure of a loss-of-function von Willebrand disease mutant of this domain.
• A detailed process for expression and purification of the p.Gly1324Ser A1 domain is described.
• Size exclusion and reverse phase chromatographic methods ensure proper protein purity and absence of protein aggregates as valuable quality control metrics.
• The crystallization methods describe the process of obtaining a crystal structure for the von Willebrand factor A1 domain harboring the loss-of-function mutation p.Gly1324Ser.
• The methods and data described establish benchmarks for obtaining high quality VWF A1 domain constructs for use in the structural and functional analysis of von Willebrand disease.

## 1 **Data**

We describe the production, inclusion body preparation, refolding and purification of the A1 domain containing the loss-of-function mutation p.Gly1324Ser. Furthermore, the data shows quality control steps that we use to ensure that the protein is sufficiently pure and natively folded. We also describe the crystallization process, data refinement and obtained parameters. Finally, the structure of the p.Gly1324Ser A1 domain variant is compared to previously published structures of the A1 domain.

## 2 Experimental design, materials and methods

### 2.1 Production of p.Gly1324Ser A1 in *Escherichia coli*

Recombinant human VWF A1 domain containing the loss-of-function mutation p.Gly1324Ser was expressed in *E. coli* M15 cells as a fusion protein containing an N-terminal 6× His Tag using BamHI and HindIII restriction sites in the Qiagen pQE-9 vector [2], [3]. For the transformation of the cells, 1 µL of plasmid was mixed with 80 µL of competent cells and heat-shocked at 42 °C for 1.5 min, followed by 1 min incubation on ice. Then 1 mL of LB-medium was added without antibiotics and the cells were incubated for 1 h at 37 °C. Six pre-cultures (25 mL of sterile LB-medium with 100 µg/mL Ampicillin and 25 µg/mL Kanamycin) were prepared by addition of 170 µL of the cell suspension and then shaken overnight at 37 °C. The following morning the pre-cultures were transferred into six flasks containing 600 mL sterile LB-medium containing 100 µg/mL Ampicillin and 25 µg/mL Kanamycin. The cultures were incubated for 2.5 h at 37 °C to reach an OD_600_ of approx. 0.6 and then protein expression was induced by addition of 1.5 mM IPTG. After 4 h the cells were harvested by centrifugation at 6000×*g*.

### 2.2 Inclusion body preparation, solubilization and refolding

Cell pellets were resuspended in 50 mM Tris HCl, 150 mM NaCl, pH 8.2 and then incubated with Lysozyme (approx. 1 mg/gram biomass) on ice. After 30 min, 4 mg/g biomass of deoxycholic acid was added and the cells were incubated at 37 °C for 10 min. For digestion of cellular RNA and DNA, RNAse and DNAse were added (0.6 mg/0.3 mg per 5 g of biomass). After approx. 30 min of incubation on a shaker, the lysed cells were centrifuged at 15,000×*g* for 15 min at 4 °C. The supernatants were discarded and the pelleted inclusion bodies were resuspended and washed in 2 M urea, 0.5% Triton X-100, 10 mM EDTA, 200 mM NaCl, 50 mM Tris HCl, and pH 8.2. This procedure was repeated three times.

The inclusion bodies were solubilized in 6 M GdnHCl, 25 mM Tris HCl, pH 7.5 at room temperature and centrifuged at 15,000×*g* for 15 min at 4 °C. The supernatant was filtered using 0.8 µm filters. Then the solubilized protein was refolded via dilution into 4 L of cold buffer containing 50 mM Tris HCl, 1 M NaCl and 0.5% Tween 20, pH 7.5.

## 3 Purification

The refolded protein was loaded overnight on a Ni NTA column, washed with 100 mM Tris HCl, 1 M NaCl, 25 mM imidazole, pH 7.5 for 3 h and then eluted with 250 mM Imidazole in the same buffer. The eluted A1 domain then was dialyzed overnight against 25 mM Tris HCl, 150 mM NaCl, 0.05% Tween 20, pH 7.5.

For the second purification step, the A1 domain was loaded on a Heparin-Sepharose column, washed with 25 mM Tris HCl, 125 mM NaCl, pH 7.5 for 3 h and eluted with 450 mM NaCl afterwards [2], [3]. The protein then was dialyzed exhaustively against TBS and stored on ice at 0 °C for a maximum of two weeks. Prior to any experiment the protein was centrifuged for at least 10 min at 60,000×g.

### 3.1 Quality control

**Fig. 1:**
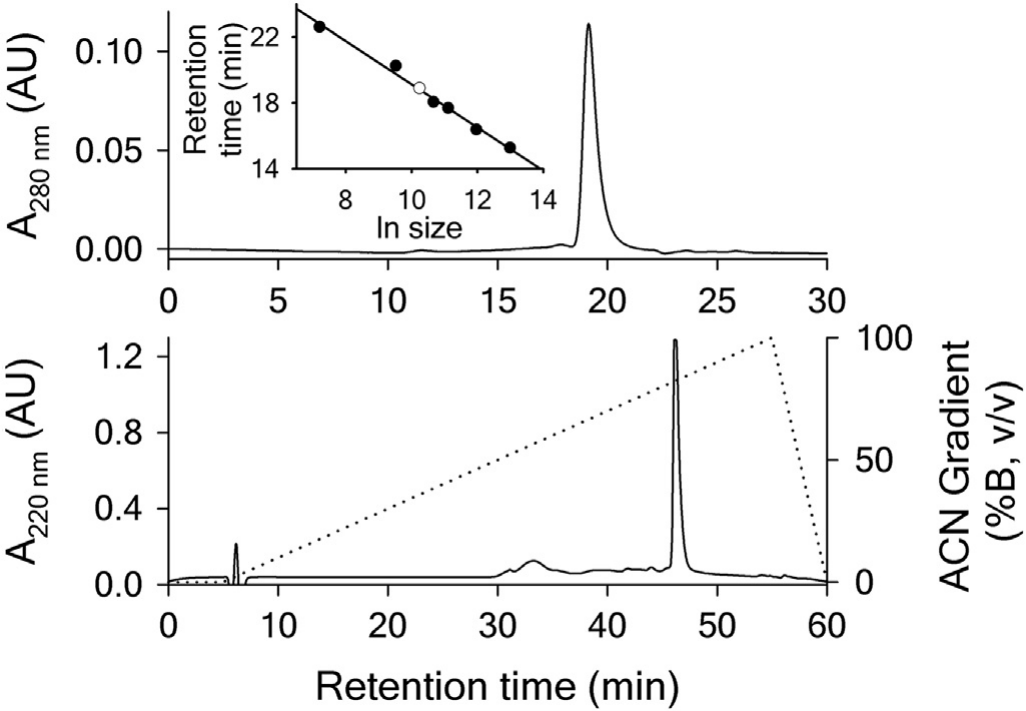
Analytical gel filtration and reverse phase HPLC of A1 p.Gly1324Ser. The upper panel shows a chromatogram of A1 p.Gly1324Ser obtained from analytical gel filtration. The inset shows a molecular weight calibration curve consisting of the retention times of Ferritin (440 kDa), Aldolase (158 kDa), BSA (67 kDa), Ovalbumin (43 kDa), Ribonuclease A (13.7 kDa) and Vitamin B12 (1.35 kDa) (●). The retention time of p.Gly1324Ser A1 is indicated by (o). The lower panel shows a reverse phase HPLC run (solid line) with a gradient (dotted line) of 2% B between 5 and 55 min.

To ensure that the protein preparation is of sufficient purity, natively folded and does not contain aggregates, reverse phase HPLC and analytical gel filtration experiments were performed. The upper panel of Fig. 1 shows an analytical gel filtration experiment performed on a Beckman System Gold Analytical HPLC System (Pump model 125, UV detector model 166) using a Phenomenex S3000 column at 0.5 ml/min. The A1 domain elutes at approx. 19 min, which demonstrates that the 29 kDa protein is monomeric and globular when comparing it with a molecular weight calibration curve (inset) obtained from retention times of Ferritin (440 kDa), Aldolase (158 kDa), BSA (67 kDa), Ovalbumin (43 kDa), Ribonuclease A (13.7 kDa) and Vitamin B12 (1.35 kDa).

Reverse phase HPLC (lower panel of Fig. 1) was performed to determine the purity of the protein preparation. An analytical Beckman HPLC system (Pump model 126, UV detector model 166) was used with a Grace Vydac C_18_ column at 0.5 mL/min. A 2% B-gradient (Buffer A: Water with 0.1% Trifluoroacetic acid, Buffer B: Acetonitrile (ACN) with 0.1% Trifluoroacetic acid) was applied, to separate the A1 domain from potential impurities. The protein elutes at approx. 45 min (70% ACN) as a single sharp peak with no other major impurities visible in the chromatogram.

## 4 Crystallization and processing

**Fig. 2:**
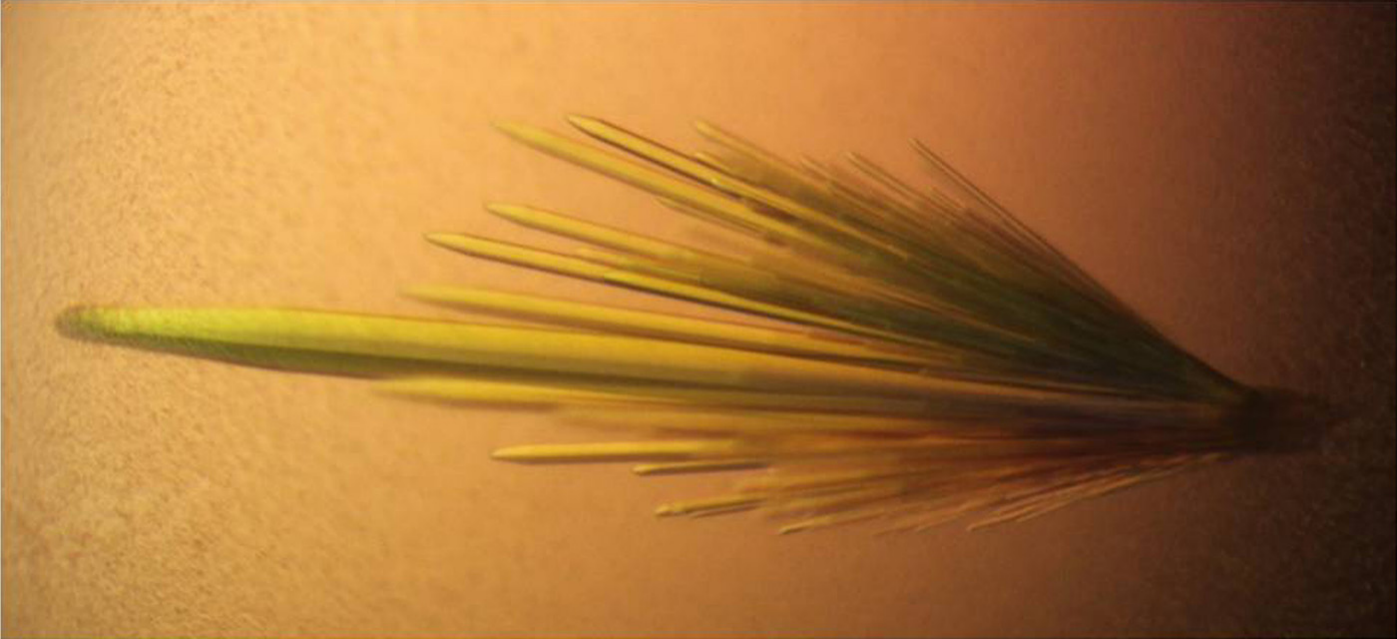
Photograph of the VWF A1 p.Gly1324Ser crystals taken with a polarized light microscope. VWF A1 p.Gly1324Ser grew as needle clusters in the presence of 30% (v/v) PEG 400, 100 mM CAPS/sodium hydroxide, pH 10.5 at 4 °C.

p.Gly1324Ser A1 was shipped on ice overnight for crystallization. The protein was then passed through a 0.22 µm filter to clear aggregates and then concentrated to 20 mg/mL. p.Gly1324Ser A1 was screened against the Wizard Classic 1 & 2 screens (Emerald BioSystems) using a mosquito Crystal (TTP Labtech). Concentrated protein was mixed 1:1 with the crystallization solutions, and set up as 400 nL hanging drops at 4 °C. The crystallization solution selected from the screen was 30% (v/v) PEG 400, 100 mM CAPS/sodium hydroxide, pH 10.5. Crystals grew within 5 days in 2 µL drops. After a five minute incubation in the crystallization solution supplemented with 33% ethylene glycol, the crystals were frozen in liquid nitrogen. Flash frozen crystals were sent to the Advanced Light Source (Berkeley, California) for diffraction experiments. Diffraction data was processed using CCP4 and iMOSFLM, CC_1/2_ values were used to guide resolution cutoffs [4], [5]. Initial phases were generated using PDB ID: 4C29 as a search model for molecular replacement (MR). MR was performed using Phenix.Phaser [6]. The model was fully refined with no Ramachandran outliers using Phenix.Refine and built using Coot [6], [7]. The data collection, processing and refinement statistics for the crystal structure are shown in Table 1 and a representative picture of the crystals is shown in Fig. 2. The structural model was deposited in the Protein Data Bank *(accession code PDB:* 5BV8).

### 4.1 Comparison of p.Gly1324Ser A1 with other crystal structures of the A1 domain

**Fig. 3:**
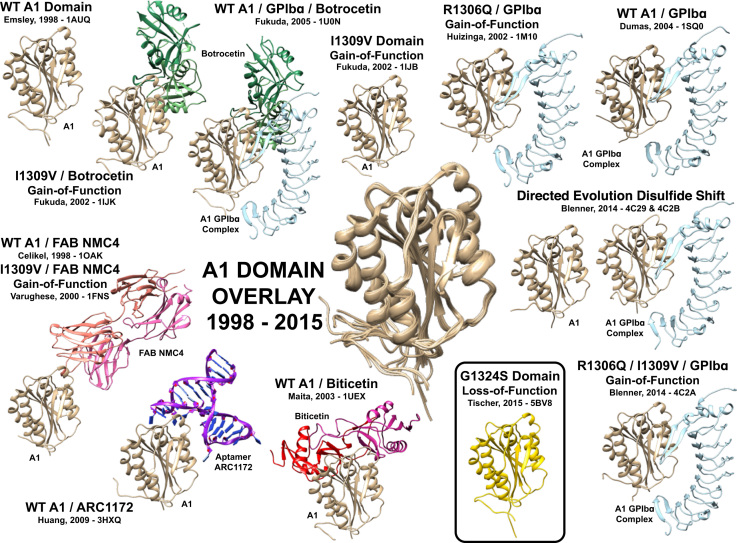
Overlay of VWF A1 domain structures available from the Protein Data Bank [8], [9], [10], [11], [12], [13], [14], [15], [16], [17]; (G1324S=p.Gly1324Ser).

Fig. 3 shows all crystal structures of the A1 domain found in the literature. Since its first crystallization in 1998 [8], the wildtype A1 domain has been crystallized in complex with the platelet receptor GPIBα [9], with the RNA aptamer ARC1172 [10], the monoclonal antibody Fab fragment of NMC4 [11] and in presence of the snake venoms Botrocetin and Biticitin [12], [13], [14]. The type 2B (gain of function) mutations I1309V and R1306 have also been crystallized either alone or in various complexes [12], [15], [16], [17]. A structure of the A1 domain where the disulfide bond has been shifted one residue towards the N-terminus alone and in complex with GPIBα has also been published [16]. The overlay, illustrated in the center of Fig. 3, compares the obtained crystal structure for p.Gly1324Ser with the structures mentioned above. Fig. 3 illustrates that p.Gly1324Ser is identical to all published structures of the A1 domain.

## Figures and Tables

**Table 1:** Crystal data, collection data, and refinement parameters for VWF A1 p.Gly1324Ser (5BV8).

**VWF A1 p.Gly1324Ser (5BV8)**			
**Data collection**		**Refinement**	
Wavelength (Å)	0.97741	Resolution (Å)	43.23–1.59
Space group	P 61	No. reflections	38781
Cell dimensions		*R*_work_/*R*_free_†	0.164/0.180
*a, b, c* (Å)	86.45, 86.45, 68.16	No. atoms	
*α*, *β*, *δ* (deg)	90, 90, 120	Proteins	1651
Resolution (Å)	68.16–1.59	Ligand/ion	5
*R*_merge_	0.081 (0.986)	Water	232
CC_1/2_‡	0.999 (0.651)	*B*-factors	
CC^*^	0.999 (0.888)	Protein	22.699
*I*/*σI*	14 (1.5)	Ligand/ion	55.408
Completeness (%)	99.9 (98.9)	Water	35.498
Redundancy	6.5 (5.7)	R.M.S. deviations	
		Bond lengths (Å)	0.006
		Bond angles (deg)	1.109

† ^5% of data were used for validation and were excluded from refinement.^

‡ CC_1/2_^is defined as the correlation coefficient between two random half data sets.^
